# Neuroinflammation in Huntington's disease: Causes, consequences, and treatment strategies

**DOI:** 10.1177/18796397251338207

**Published:** 2025-08-07

**Authors:** Alina Blusch, Maria Björkqvist

**Affiliations:** 1 Wallenberg Neuroscience Center, Brain Disease Biomarker Unit, Department of Experimental Medical Science, Lund University, Lund, Sweden

**Keywords:** Huntington's disease, neuroinflammation, inflammation, immune cells, immune system, treatment strategies

## Abstract

Huntington's disease (HD) is a progressive neurodegenerative disorder, and increasing evidence suggests that inflammation, both central and peripheral, plays a role in disease progression. Neurohistology and neuroimaging studies illustrate neuroinflammatory processes as part of HD pathophysiology. Furthermore, studies of blood and cerebrospinal fluid from HD patients show altered levels of inflammatory markers and immune cell populations that could influence neuroinflammation and the neurodegenerative process. Here, we review findings contributing to our understanding of the significance of immune activation in HD pathology. We discuss evidence of intrinsic effects of mutant huntingtin within immune cells and central immune alterations that contribute to neuroinflammation and neurodegeneration. We address the roles of central immune cells, as well as the potential contributions of peripheral signals and cell types in HD immune activation. We further discuss opportunities and challenges in utilizing immune-modulation strategies for future treatment approaches. A better understanding of neuroimmune interactions in HD can provide insights for manipulating these responses, potentially facilitating the development of therapies aimed at reducing the impact of neuroinflammatory and degenerative processes.

## Introduction

Traditionally considered an immune-privileged organ, the brain is now recognized to harbor a unique and specialized set of resident immune cells.^1^ The role of inflammation in the brain is complex, with ongoing debates about whether reducing inflammation could slow disease progression or inadvertently worsen it. Chronic inflammation, for example, is known to be neurotoxic, yet it also plays a crucial role in the brain's natural repair processes. Neuroinflammation refers to the brain's physiological response to various stimuli, such as injury, infection, stress, or aging, which activates resident immune cells like microglia (the brain's innate immune macrophages) and astrocytes.^2^

While the systemic immune system is essential for defending the body against injury or infection, it can also contribute to chronic systemic inflammation, which may, in turn, promote neuronal damage in neurodegenerative diseases.^3^ Age-related changes further complicate this immune response. Aging is a significant risk factor for neurodegenerative diseases and leads to a persistent, low-grade inflammatory state.^4^ Despite the heterogeneity of neurodegenerative diseases, chronic low-grade inflammation and systemic immune activation are common features across conditions.^5^ The concept of neuroinflammation as a contributor to neurodegenerative disease pathology was first proposed in the 1940s. Since then, extensive research has confirmed the involvement of inflammation in the pathogenesis of several neurodegenerative disorders, including Alzheimer's disease (AD), Parkinson's disease (PD), Amyotrophic lateral sclerosis (ALS), and HD, with neuroinflammation now recognized as a key mechanism underlying neurodegeneration.^5–7^ In HD, accumulating evidence underscores the role of neuroinflammation in the disease's progression. Both neurohistological and neuroimaging studies have demonstrated neuroinflammatory processes at various stages of the disease, from early to late stages, highlighting its potential impact on disease progression (reviewed in^2,8,9^). A common characteristic of neuroinflammation across neurodegenerative disorders is the central activation of microglia and astrocytes, alongside a low-grade systemic immune response, typically associated with elevated levels of pro-inflammatory cytokines. In AD,^10,11^ PD,^12^ and ALS,^13^ these inflammatory processes are well-documented. Similarly, in HD, a low-grade systemic immune response is detectable even before the onset of clinical symptoms.^14^ Studies of peripheral blood and cerebrospinal fluid (CSF) from HD patients further suggest alterations in inflammation-related biomarkers and immune cell populations, which may contribute to the neuroinflammatory processes and accelerate neurodegeneration.^15,16^

This review will focus on neuroinflammation in HD, exploring its potential contributions to neuropathology, the key cell types involved, and the timing and location of neuroinflammatory processes. We will discuss the complexity of the immune response in HD, including the effects of mutant huntingtin in immune cells and the broader extrinsic immune responses. A deeper understanding of central immune activation and the interactions between the central and peripheral immune systems in neurodegenerative diseases, particularly HD, is critical for developing immune-modulation strategies that could serve as disease-modifying therapies.

## Which immune cells are involved?

**Figure 1.**
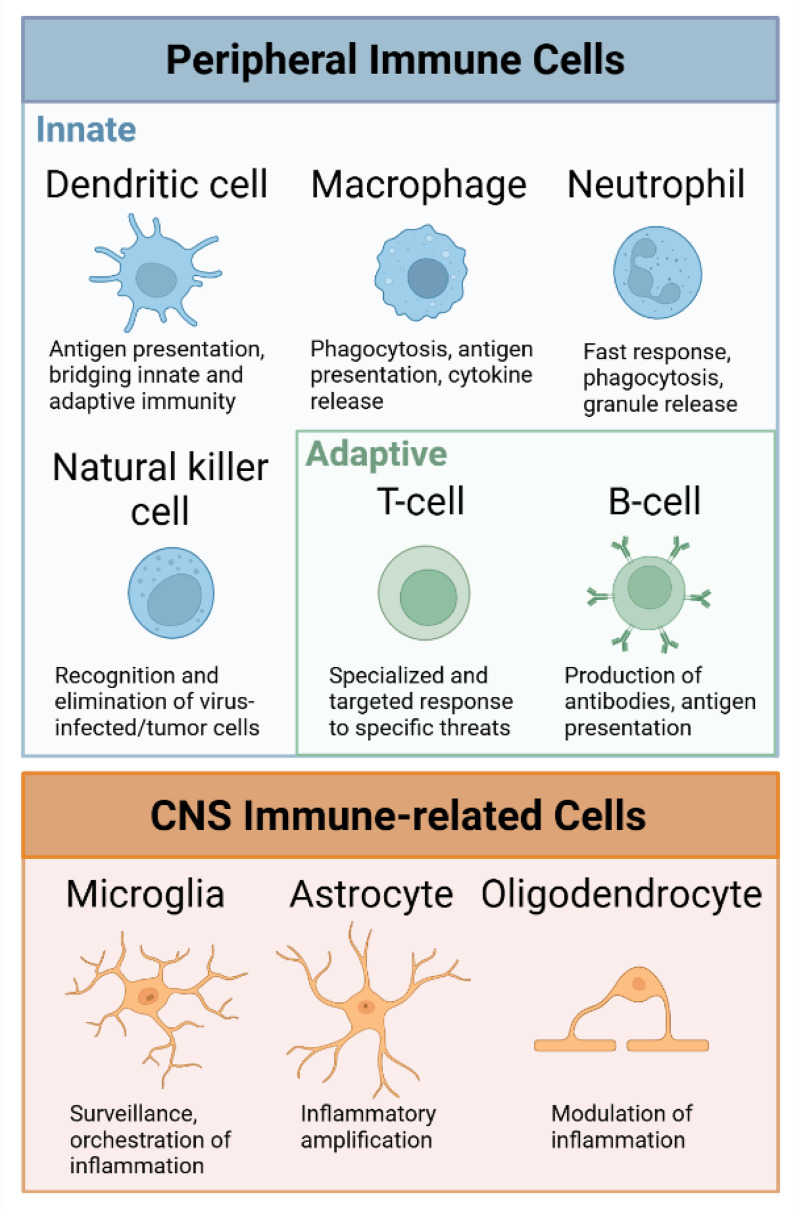
Short overview of immune cells involved in the immune response in Huntingtońs disease. Peripheral immune cells contribute to the immune response in neurodegeneration, with dendritic cells, macrophages, neutrophils, and natural killer cells representing the innate immune system, while T cells and B cells belong to the adaptive immune system. In the CNS, the major cell types contributing to inflammatory processes are microglia, astrocytes and oligodendrocytes. Created with Biorender.com.

The role of immune cells in HD is a subject of growing interest. Huntingtin expression is high in neurons compared to other cell types. Importantly, HD subject immune cells, such as monocytes, macrophages, and B and T cells express mutant huntingtin, which leads to dysfunction characterized by excessive inflammatory cytokine production and altered immune signaling pathways^17^ correlating with disease status.^18,19^ Immune reactions in HD appear to result from a combination of extrinsic effects, driven by ongoing neurodegeneration, and intrinsic factors where mutant huntingtin directly alters the behavior of immune cells. Evidence suggests that mutant huntingtin exerts both direct and indirect effects on inflammatory cells, contributing to the interplay between neurodegeneration and immune dysfunction in HD (reviewed in^9,20^). The primary cell types involved in inflammatory processes in the brain can be categorized into innate immune cells, adaptive immune cells, and central nervous system (CNS) immune-related cells (for a brief overview of immune cells see Figure 1).

The expression of mutant huntingtin within glial cells plays a significant role in the exacerbating neurotoxicity.^21^ Several studies indicate that microglia and astrocytes are the primary cell types driving neuroinflammation in HD.^20^ Microglia have been demonstrated to be key drivers of neuroinflammation in HD, with early activation detectable in presymptomatic gene carriers up to 15 years before predicted onset, which correlates with disease severity and neuronal loss in affected regions,^22,23^. The prevalence of mutant huntingtin in microglia may be attributed to their enhanced capacity for clearing misfolded proteins compared to neurons, which exhibit lower efficiency in proteostasis mechanisms. Additionally, microglia themselves express mutant huntingtin, which may directly affect inflammation in the CNS.^24^ A study by Petkau et al. demonstrated that knockdown of mutant huntingtin in BACHD microglia did not alleviate HD-related behavioral deficits or neuropathology and neuroinflammation. In contrast, another study indicated that the hyperactive release of pro-inflammatory cytokines, such as IL-6, by stimulated YAC128 microglia is intrinsically driven, with suppression of mutant huntingtin normalizing IL-6 release.^25^ HD microglia exhibit elevated production of pro-inflammatory cytokines even under basal conditions, along with impaired phagocytosis and endocytosis capabilities.^26^ Microglia have also been suggested to play a role in synaptic alteration and loss during HD pathogenesis,^27,28^ and depletion of microglia has been shown to prevent striatal volume reduction as well as phenotypic alterations in the R6/2 mouse model.^29^

CNS injury and disease, along with activated neuroinflammatory microglia induce reactive astrocytes.^30^ Reactive astrocytes are abundant in the HD brain and astrocytes have been demonstrated to contain mutant huntingtin in human HD, as well as in mouse models of the disease.^31^ In BACHD mice, expression of mutant huntingtin in astrocytes has been shown to be necessary for HD pathogenesis, as reduced expression slowed the progression of the behavioral and neuropathological phenotypes.^32^ Astrocytes play a significant role in HD-related inflammation as they become reactive, release pro-inflammatory cytokines and neurotoxic molecules, disrupt glutamate and ion homeostasis, and sustain the inflammatory response in collaboration with microglia.^31,33^ They undergo activation locally in response to dead neurons or pathogens, and the presence of mutant huntingtin in astrocytes can also diminish their neuroprotective functions.^34^ As microglia, activated astrocytes contribute to the increased production of inflammatory mediators.^16,35^

Oligodendrocytes play a critical role in neuroinflammation by expressing inflammatory mediators, which enables them to sense and respond to inflammation while also influencing microglial phenotype through their wide-ranging immunomodulatory properties.^36^ In HD, oligodendrocytes and oligodendrocyte precursors are arrested at intermediate maturation stages, with altered expression of inflammatory markers, as shown in the R6/2 mouse model and human HD post-mortem tissue.^37^

Cells from both the innate and adaptive immune systems, including T cells, B cells, and myeloid cells such as monocytes and macrophages, play a role in the immune response associated with neurodegeneration. Additionally, peripheral immune cells have been shown to contribute to HD pathology. Human HD myeloid cells produce more inflammatory cytokines due to cell-intrinsic effects of mutant huntingtin expression linked to a direct effect on the NFκB pathway. By lowering huntingtin expression, the myeloid cell function was normalized.^17^ Human HD monocytes exhibit transcriptional differences, such as increased IL-6 expression under both basal and stimulated conditions.^38^ In human HD as well as in HD mouse models, monocytes and macrophages show increased activation and likely contribute to the increased levels of cytokines detected in blood and CSF.^39^ Both peripheral and CNS immune system activity has been shown to increase with disease progression in HD mouse models. Importantly, depletion of monocytes and macrophages in vivo show that these cells play a central role in the pathogenic immune response.^19^

Adaptive immune cells, particularly T cells, have been implicated in the inflammatory processes of HD.^40^ However, it has also been noted that the influx of peripheral immune cells—such as lymphocytes and neutrophils—into the brain is rare in HD.^41^ Elevated levels of soluble CD27, a marker of T cell activation, and an increased prevalence of TH17.1 cells have been observed in the CSF of premanifest HD patients.^40,42^ Additionally, subsets of regulatory T cells and Th2 cells may exert beneficial effects in HD, potentially through the production of brain-derived neurotrophic factor (BDNF) or interleukin-4 (IL-4).^43^ T cells might also play a role in the altered cytokine profile seen in HD, particularly in the later stages of the disease. For instance, HD mutation carriers exhibit significantly higher levels of IL-4 in plasma compared to controls, suggesting the involvement of Th2 cells in the disease process.^14^

Disruption of the blood-brain barrier (BBB) and immune cell infiltration are hallmarks of several neurodegenerative diseases. In HD, while evidence of BBB disruption is limited, some studies indicate changes in BBB integrity. Increased BBB permeability has been observed in HD iPSC-derived brain microvascular endothelial cells, and another study demonstrated that in R6/2 mice, BBB disruption occurs early in disease progression.^44,45^ Pericytes are part of the BBB and are involved in mediating neuroinflammatory responses, which are known to be altered in HD. Interestingly, an increase in activated pericytes has been demonstrated both in the R6/2 brain and in postmortem HD brain tissue.^46^ Moreover, a study shows age-dependent neurovascular alterations and changes in microglial morphology in YAC128 brain, further underscoring the neurovascular dysregulation observed in HD.^47^ This involvement suggests that pericyte dysfunction may contribute to disease progression and potentially to neuronal loss, illustrating the complexity of the immune response in HD.

## When and where? Timing and spatial considerations in HD neuroinflammation

**Figure 2.**
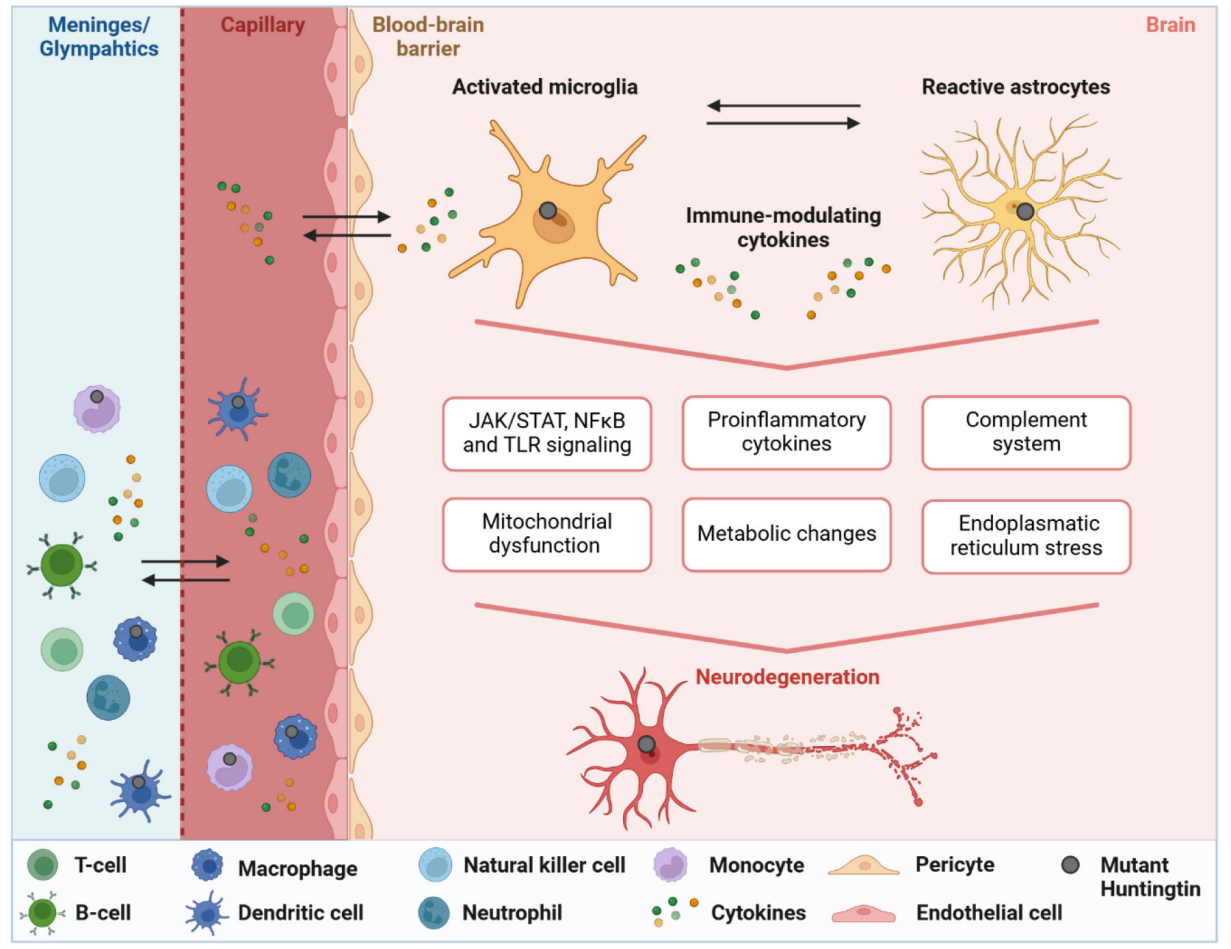
Studies in both human Huntington's disease (HD) and HD mouse models reveal a complex and multifaceted inflammatory response. Neuroinflammation in HD is characterized by disrupted microglial-neuronal interactions, resulting from the activation of microglia and astrocytes. Mouse models typically show elevated levels of pro-inflammatory cytokines such as IL-6 and TNF-α, but human studies present a more intricate and varied inflammatory profile. These discrepancies underscore the challenges of translating findings from animal models to human HD. Pro-inflammatory cytokines, activation of the complement system, and signaling through pathways such as JAK/STAT, NF-κB, and Toll-like receptors (TLRs) play a central role in driving neuroinflammation and neuronal damage. Additionally, metabolic disturbances, including mitochondrial dysfunction and chronic endoplasmic reticulum stress, contribute to neurodegeneration. Bidirectional cytokine exchange between the capillaries and the brain serves as a key mechanism for communication between the central nervous system and the peripheral immune system. The exchange of cytokines and immune cells between the meninges/glymphatic compartment (depicted as one due to simplification), an important neuroimmune interface for immune surveillance and protection, and the circulating blood appears to play a role in HD, though it remains understudied. Accumulating results highlight the importance of developing early interventions that target inflammation in HD. Created with Biorender.com.

The timing of inflammation in HD is an aspect to consider, as inflammatory processes have been shown to begin early in the disease course, even before clinical symptoms appear.^14,22,48^ In mouse models, inflammatory changes can be detected long before motor symptoms develop, with inflammation increasing steadily as the disease progresses.^49,50^ However, a recent study on HD gene carriers in the HD-ISS stage 0 found no significant signs of inflammation, as pro-inflammatory cytokines IL-6 and IL-8 exhibited unchanged longitudinal levels. The authors suggest that astrocytic dysfunction is more prominent than any abnormal innate immune response at this early stage, further supporting the idea that neuroinflammation does not significantly manifest until later stages of the disease.^51^ Do we currently know enough about early immune dysfunction and how (and if) it alters along disease course? As evidence suggests that mutant huntingtin exerts both direct and indirect effects in and on inflammatory cells, this contributes to the complex interplay between neurodegeneration and immune dysfunction in HD (reviewed in^9,20^). The intrinsic impact of mutant huntingtin within myeloid cells may indicate that immune cell activation and the resulting immune alterations lead to a minimal and persistent chronic inflammation in HD. Possibly, as the disease progresses, there are ongoing processes that result in additional immune responses (Inflammatory processes in HD are illustrated in Figure 2, providing a broad overview of the underlying mechanisms).

An ongoing question in HD research is whether brain inflammation occurs in specific regions or if it is a broader feature. Imaging studies consistently identify prominent inflammatory changes in areas such as the basal ganglia and striatum.^8,20,52^ Even in premanifest HD gene carriers, PET imaging has shown microglial activation in the cortex, basal ganglia, and thalamus.^22^ In HD patients, increased expression of key inflammatory mediators has been observed in the striatum and cortex, and, somewhat unexpectedly, in the cerebellum.^41^ However, certain brain regions have received less attention or show minimal evidence of inflammation. For example, regions such as the hypothalamus, amygdala, and hippocampus—each of which undergoes atrophy in HD—have not been extensively studied for signs of neuroinflammation. It is important to note that the hypothalamus is central to the brain-body immune communication and the action of cytokines. For instance, it influences metabolism by acting on hypothalamic nuclei involved in feeding and homeostasis.^53^ Similarly, several adjacent structures, including the meninges, glymphatic system, and choroid plexus, play critical roles in neuroinflammatory processes but have been understudied in the context of HD. Recent research has increasingly recognized the importance of these structures in regulating both local and systemic inflammation within the brain.^54,55^ Impairment of the glymphatic system is a common feature across neurodegenerative diseases. It is thought that glymphatic system impairment leads to the accumulation of proteins and waste products, which in turn triggers inflammation.^56^ A recent study has specifically highlighted disturbances in the glymphatic network in HD, with dynamic glucose-enhanced MRI revealing a significant reduction in CSF clearance efficiency before the phenotypic onset of the disease that worsened with disease progression.^57^ Moreover, glymphatic dysfunction poses a challenge for the effective distribution of antisense oligonucleotides within the brain. Modulating glymphatic activity may thus enhance the delivery and potency of antisense oligonucleotides in HD treatment, making it a promising avenue for therapeutic exploration.^58^ Concerning the mechanisms by which mutant huntingtin is cleared from the brain, research has demonstrated that huntingtin enters the CSF through both passive release and active secretion, followed by glymphatic clearance in mice.^59^ Future studies are likely to elucidate the potential of glymphatic clearance as an early biomarker and a viable therapeutic target for HD. Similarly, the meninges, once considered a mere structural component, have now been recognized for their crucial role in maintaining immune homeostasis and managing inflammation, implicating them in the development of neurological disorders.^60^ The lack of data on the brain regions and structures mentioned above does not imply that they are unaffected by inflammation, but rather points to the need for further research to understand the extent and impact of inflammation in these areas and its potential connection to neurodegeneration in HD.

These spatial aspects of inflammation reveal both commonalities and differences between human and mouse models of HD.^8,61^ The presence of the mutated huntingtin protein is thought to set the stage for early, subtle inflammation, potentially involving low-grade immune activation. As the disease progresses, there seems to be a more amplified immune response with pronounced inflammation. Studies in both mouse models and human HD have investigated early and late inflammatory processes in HD, showing that inflammation is present even before symptom onset, though its extent varies across studies, highlighting the complexity of immune activation throughout disease progression. Human studies tend to show more heterogeneous cortical involvement, while mouse models provide more detailed insights into specific regions and molecular mechanisms. These differences should be kept in mind when interpreting findings from HD studies and considering the broader implications of inflammation in the disease.

## How? Signaling pathways, interplay, and complexity of the immune response in HD

The brain's communication with the immune system is essential for maintaining neural health and overall homeostasis. In neurodegenerative diseases, interactions between immune cells and neurons, as well as metabolic exchanges, play a significant role.^62^ Key communication modes, including cytokine signaling and the complement system, mediate inflammation and influence both immune function and brain integrity.

Neuroinflammation and activation of microglia are present in premanifest HD gene carriers^48^ and are mirrored peripherally by activated monocytes and increased levels of plasma cytokines.^14^ The levels of immune markers in the CSF and peripheral blood may reflect the immune pathology associated with HD, and the expression of mutant huntingtin within immune cells. Several studies have been conducted that focus on immune markers in biofluids. The results of these studies have indicated a correlation between levels of immune markers in blood, CSF or saliva and disease progression.^14,63–66^ Cytokine signaling pathways play a central role in immune dysregulation. Collectively, research suggests that disturbances in both innate and adaptive immune systems, in the brain and periphery, lead to the upregulation of inflammatory cytokines and signaling markers.^14,63–66^ Immune responses may vary with disease stage, showing distinct early and late characteristics. Early responses may focus on protective and regulatory functions, while later responses often become more dysregulated, potentially exacerbating inflammation and neuronal damage. This variation in immune responses raises the important question: which major immune pathways and signaling mechanisms are affected in HD?

During homeostasis, basal and stable activity of inflammatory pathways is maintained, whereas a disturbance of these pathways is linked to chronic inflammation. Immune signaling pathways such as the JAK/STAT and NFκB signaling pathways are important under both healthy and neurodegenerative conditions. Research has demonstrated that the hyperreactivity of HD monocytes and microglia is linked to dysregulation of the NFκB pathway.^17^ NFκB is a central modulator of inflammatory responses and plays a significant role in regulating the expression of pro-inflammatory cytokines (reviewed in^67^). Additionally, the JAK/STAT pathway, particularly STAT3, is aberrantly activated in HD, promoting the expression of pro-inflammatory genes and contributing to chronic neuroinflammation.^68^ This pathway plays a role in glial activation, increasing the production of inflammatory mediators by astrocytes and microglia. Similarly, constitutive activation of the NFκB pathway in HD may drive persistent inflammation through the production of pro-inflammatory cytokines and chemokines. These pathways often work in tandem, with mutual activation creating a feed-forward loop that amplifies the inflammatory response in HD.

Toll-like receptors (TLRs) are pattern recognition receptors involved in the immune response to pathogens. All major CNS cell types express toll-like receptors, particularly TLR4, which is crucial for immune functions. Alterations in TLR4 activation can contribute to neuroinflammation and neurodegeneration.^69^ In HD, activation of TLRs by endogenous damage-associated molecular patterns (DAMPs) could promote chronic neuroinflammation. Overactivation of TLRs in microglia triggers the release of pro-inflammatory cytokines, and contributes to sustained glial activation and neurodegenerative processes in HD. Low expression of TLR4 has been shown to exert beneficial effects on neuropathology and motor phenotype in an HD mouse model.^70^

The complement system, a key component of the innate immune response, also plays an important role in regulating normal CNS function. However, when activated in the brain, complement proteins can contribute to microglial activation, cytokine release, and chronic neuroinflammation, which may exacerbate neurodegenerative processes.^71^ Recent studies have highlighted the complement system's involvement in modifying the progression of HD. In HD patients, selective loss of synaptic connections between the cortex and striatum, observed in postmortem tissue, correlates with increased activation and localization of complement proteins, particularly C3 and C1q.^28^ Elevated levels of complement proteins, including C3 and C1q, have been detected in the CSF of premanifest HD patients, highlighting the involvement of complement activation in the disease process.^28^ It is also important to note that the complement factor C1QB did not exhibit significant changes in the CSF of HD patients.^72^

Additionally, the NOD-like receptor protein 3 (NLRP3) inflammasome plays a critical role in mediating innate immunity and inflammation by activating caspase-1 and inflammatory cytokines (reviewed in^73^). In an HD mouse model, suppressing the NLRP3 inflammasome has shown beneficial effects.^74^

In summary, substantial evidence indicates that activation of the innate immune system is present in HD, characterized by microglial activation and expression of pro-inflammatory cytokines, as well as impaired migration of macrophages. Moreover, studies have demonstrated the involvement of an adaptive immune response in HD with dendritic cells that prime T-cell responses. Potentially both innate and adaptive immune responses are important for HD pathogenesis (reviewed in^43^).

## More factors adding to the complexity of neuroinflammation

Several factors contribute to the complexity of immune responses, including aging, metabolism, gut-microbiota and endoplasmic reticulum (ER) stress. Age is a well-established risk factor for neurodegenerative diseases, and it is also associated with changes in immune function. Specifically, aging is linked to alterations in both the innate and adaptive immune responses, often manifesting as a chronic, low-grade inflammation referred to as “inflammaging”.^4^ The brain, which has a high energy demand, is particularly vulnerable to mitochondrial dysfunction.^75^ Mitochondrial impairment has been implicated in the pathophysiology of HD, as it reduces energy production and promotes the generation of reactive oxygen species.^76,77^ Research in other neurodegenerative disorders indicates that mitochondrial deficits in microglia contribute to their inflammatory phenotype. Dysfunctional mitochondria intensify inflammasome-mediated pro-inflammatory signaling, creating a self-perpetuating cycle of inflammation and mitochondrial damage that exacerbates neurodegeneration.^78,79^ In HD, mitochondrial dysfunction leads to decreased ATP production, resulting in energy deficits that heighten neuronal vulnerability.^76,77^ A study revealed mitochondrial dysfunction in YAC128 astrocytes and highlighted the modulation of this dysfunction by the oxidative stress response proteins HACE1 and Nrf2.^80^ Additionally, impaired glucose metabolism may further exacerbate neurodegeneration in HD (reviewed in^81^). Gut microbiota alterations and metabolic changes have been suggested to contribute to inflammation and the pathogenesis of HD.^82^ Recent studies indicate that gut dysbiosis contributes to systemic inflammation and neurodegeneration via the gut-brain axis.^83^ Increased intestinal permeability allows bacterial translocation and inflammatory mediators to enter circulation, potentially worsening neuroinflammation. Additionally, microbiome alterations impact neurotransmitter and metabolite production, highlighting a bidirectional gut-brain communication essential for neurological health and disease progression.^83^ The cytokines and chemokines released by adipose tissue can cross the blood-brain barrier, activating microglia and further amplifying CNS inflammation.^84^ Furthermore, chronic ER stress exacerbates both inflammation and neuronal damage, playing a pivotal role in the progression of neurodegeneration.^85^ In HD, the accumulation of misfolded proteins, particularly mutant huntingtin, triggers ER stress, activating the unfolded protein response.^86^ This response can also promote inflammation through the release of inflammatory cytokines.

## Neuroinflammation as a potential target for disease modification in HD

The observable immune response in HD and the growing recognition that immune dysfunction may contribute to disease pathogenesis have led to a significant body of research exploring potential disease-modifying strategies (reviewed in^87–89^). Several approaches have been investigated to target different aspects of immune activation, with a particular focus on microglia and other immune cells (see Table 1 for a summary of anti-inflammatory strategies and outcomes). Minocycline, a tetracycline-derived antibiotic traditionally used to treat infections,^90^ has been shown to inhibit microglial activation and reduce phagocytic activity.^91^ Despite its preclinical promise, a Phase 2/3 trial conducted by the Huntington Study Group from 2006 to 2008 found no significant clinical improvements, leading to the discontinuation of further studies.^92^ Laquinimod, an immunomodulatory drug, has demonstrated effects in reducing pro-inflammatory cytokine production in peripheral blood mononuclear cells and modulating neuroglial activation in the brain. In HD mouse models, Laquinimod has exerted beneficial immunomodulatory effects and has been shown to mitigate mutant huntingtin-induced dysfunction in oligodendrocytes,^93–95^. Although the exact mechanism of action remains unclear, Laquinimod has been shown to dampen hyperactive cytokine production in isolated HD monocytes.^96^ Despite promising preclinical data, a Phase 2 clinical trial did not demonstrate efficacy in HD as no improvements were observed in motor symptoms assessed by the Unified Huntington's Disease Rating Scale Total Motor Score (UHDRS-TMS). However, Laquinimod treatment showed beneficial effects by reducing volume loss in the caudate and other brain regions in patients with early HD.^97^ Short-chain fatty acids are key products of gut bacterial fermentation, and have been shown to modulate immune responses in neuroinflammatory diseases. A recent study examined the anti-inflammatory effects of the short-chain fatty acid propionate in the R6/2 mouse model. While propionate treatment resulted in a mild reduction of inflammatory markers in the CNS, evidenced by decreased SPI1-mRNA levels, fewer iNOS-positive cells, and normalized TNFα-mRNA levels in the motor cortex, it did not confer significant protective effects on neuronal function or alter the clinical progression of the disease.^98^ Propionate's ability to exert benefits through dietary interventions, such as fiber-rich diets, underscores its practical applicability in therapeutic approaches. Elevated levels of IL-6 are consistently observed in HD, both centrally and peripherally, prompting investigations into whether targeting IL-6 could offer therapeutic benefits. However, studies using IL-6 deficient R6/2 mice found that these animals exhibited a more severe phenotype, suggesting that IL-6 may play a protective role.^99^ In contrast, treatment with etanercept, an anti-TNF-α strategy, lowered plasma levels of several pro-inflammatory cytokines in R6/2 mice, leading to a deceleration of brain atrophy, although motor and cognitive functions were not significantly affected.^19^ A study found that systemic anti-huntingtin immunotherapy rescued the hyperactive release of IL-6 in stimulated peripheral YAC128 macrophages.^100^ Furthermore, research conducted in 2016 on P110, an inhibitor of pathological mitochondrial fragmentation, demonstrated that this treatment reduced circulating levels of IL-6 and TNF-α, suggesting potential for mitigating neuroinflammation in HD.^101^ Neflamapimod, an inhibitor of p38 mitogen-activated protein kinase alpha (p38 MAPK), has been shown to inhibit IL-1β and TNF-α release in peripheral blood mononuclear cells^102^ and shift microglial activation from a pro-inflammatory to a phagocytic state in animal models.^103^ A 2019 clinical study, aimed at evaluating the safety and efficacy of Neflamapimod (VX-745) in HD patients, was terminated early before completion. The study was halted in 2020 due to delays related to COVID-19.^104^ Astrocyte dysfunction is another potential therapeutic target, with the SEMA4D inhibitor Pepinemab showing mixed results in a Phase 2 trial for HD.^105^ The primary endpoints of the Pepinemab clinical trial were related to cognitive assessments and clinical global impression of change, with neither meeting the statistical threshold for success of the study. However, treatment with Pepinemab resulted in significantly greater cognitive improvements in early-stage HD patients, and the therapy also significantly reduced caudate shrinkage.

**Table 1.** Anti-inflammatory strategies evaluated in HD.

Target/compound Mechanism in short	Mouse (outcome and reference)	Human (outcome and reference)
*Glial Cells Activation / Astrocyte Dysfunction*		
Inhibition of SEMA4D SEMA4D plays an important role in activation of glial cells^108^	⇓striatal and cortex atrophy ⇑cognitive and behavioral deficits^109^	Pepinemab, mixed results (Phase 2 trial, Signal-HD)^105^
*Immune Modulation / Microglia Activation*		
Minocyclin Inhibits microglial activation, reduces phagocytic activity of microglia and macrophages		No improvement (Huntington Study Group DOMINO Investigators 2010)^92^
Laquinimod Reduces cytokine production in immune cells and microglia activation	⇓neuronal caspase-6^93^ ⇑motor function^94^	⇓cytokine production in isolated HD monocytes^96^ No improvement (LEGATO-HD, clinicaltrials.gov ID: NCT02215616)^110^
Neflamapimod Inhibitor of p38 MAPK, inhibits IL-1β and TNF-α release in peripheral immune cells and reduces microglial activation		No improvement (clinicaltrials.gov ID: NCT03980938)^111^
Galactin-3 suppression Reduction of microglia activation	⇓microglial-mediated inflammation^112^	
Propionate Suppresses pro-inflammatory cytokines and promotes regulatory T cell differentiation	⇓ inflammatory factors^98^	
*Cytokine Inhibition*		
TNF-alpha inhibition	⇓mHtt aggregates ⇑neuronal density, improved motor function^113^	
IL-6 inhibition	⇓weight, motor deficits^114^ worsened phenotype^99^	
*Complement Activation*		
Inhibition of C1q	⇓pathological synapse loss ⇑cognitive function^28^	ANX005, humanized anti- C1q antibody. Phase 2 study, primary outcomes met (clinicaltrials.gov ID: NCT04514367)^115^
Removing C3 receptor on microglia	⇓pathological synapse loss ⇑cognitive function^28^	

Researchers have explored strategies to inhibit complement activation as a potential therapeutic avenue for HD. Elevated levels of complement proteins, such as C3 and iC3b, have been linked to synaptic loss and neuroinflammation, suggesting potential avenues for therapeutic intervention. In animal models, complement inhibition has shown promise. Wilton and colleagues demonstrated that targeting complement-dependent pathology in HD mice (q175 model) with either a C1q function-blocking antibody or genetic ablation of a complement receptor on microglia prevented pathological synapse loss and associated cognitive deficits.^28^ These findings suggest that complement inhibition may represent a viable therapeutic strategy in HD. A phase 2 study utilizing a humanized anti-C1q antibody was conducted in 2020, the drug was reported well tolerated and primary safety outcomes were met.^106^ Stabilization of disease progression occurred in the overall HD patient population, with clinical benefits observed in patients exhibiting higher baseline complement activity. The presence of mutant huntingtin in microglia suggests that reducing its levels within these cells could help mitigate microglial activation. However, current studies present conflicting findings on this potential approach. In BACHD mice, a strategy involving the depletion of mutant huntingtin specifically in microglia using Cre recombinase did not rescue the HD-associated phenotype.^107^ In contrast, microglia depletion in R6/2 mice led to a reduction in striatal atrophy and a decrease in mutant huntingtin accumulation.^29^

The findings of clinical anti-inflammatory treatments that have been explored thus far remain inconclusive. This is likely due to the influence of multiple factors. Further research is necessary to determine whether these therapeutic interventions might be more effective at certain stages of the disease. However, determining the optimal timing for such treatments remains challenging. The immune response in HD is subtle, which raises the question whether immune modulation or anti-inflammatory strategies will have the capacity to affect clinical outcomes in isolation, or whether they are more likely to constitute a component of a combination therapy. Additionally, a deeper understanding of immune alterations in HD could help identify patient subgroups that might benefit from specific immune-modulatory therapies.

## Conclusion and future directions

While the precise molecular mechanisms remain unclear, mounting evidence suggests that immune activation may have an impact on the progression of HD pathology. Neuroinflammation is complex, involving a range of cells and pathways, and both the intrinsic effects of mutant huntingtin in immune cells and their responses to external factors appear to contribute to the disease. The immune response can have dual effects: it can be protective, aiding in cellular repair, or detrimental, exacerbating damage under certain conditions. It remains uncertain whether reducing inflammation would slow disease progression or inadvertently worsen it, and it is possible that the immune response may initially be beneficial but become harmful over time. Although inflammation is recognized as a factor in HD progression, targeting it therapeutically has proven challenging. Anti-inflammatory treatments have been explored as potential therapeutic options, but their effectiveness remains inconclusive. Ultimately, further research is needed to better understand the role of inflammation in HD and to evaluate whether anti-inflammatory strategies could serve as effective treatment options.
